# Regulation of Photosystem II Heterogeneity and Photochemistry in Two Cultivars of C_4_ Crop Sugarcane Under Chilling Stress

**DOI:** 10.3389/fpls.2021.627012

**Published:** 2021-02-10

**Authors:** Sonal Mathur, Valiaparambil Sebastian John Sunoj, Nabil Ibrahim Elsheery, Vangimalla R. Reddy, Anjana Jajoo, Kun-Fang Cao

**Affiliations:** ^1^State Key Laboratory of Conservation and Utilization of Subtropical Agro-bio-resources, Guangxi Key Laboratory of Forest Ecology and Conservation, College of Forestry, Guangxi University, Guangxi, China; ^2^School of Life Sciences, Devi Ahilya University, Indore, India; ^3^Adaptive Cropping Systems Laboratory, U.S. Department of Agriculture-AgriculturalResearch Service (USDA-ARS), Beltsville Agricultural Research Center, Beltsville, MD, United States; ^4^Department of Agricultural Botany, Tanta University, Tanta, Egypt; ^5^School of Biotechnology, Devi Ahilya University, Indore, India

**Keywords:** chlorophyll *a* fluorescence, chilling stress, photosynthesis, PSII heterogeneity, sugarcane

## Abstract

In subtropical regions, chilling stress is one of the major constraints for sugarcane cultivation, which hampers yield and sugar production. Two recently released sugarcane cultivars, moderately chilling tolerant Guitang 49 and chilling tolerant Guitang 28, were selected. The experiments were conducted in the controlled environment, and seedlings were exposed to optimum (25°C/15°C), chilling (10°C/5°C), and recovery (25°C/15°C) temperature conditions. PSII heterogeneity was studied in terms of reducing side and antenna size heterogeneity. Under chilling, reducing side heterogeneity resulted in increased number of Q_B_ non-reducing centers, whereas antenna side heterogeneity resulted in enhanced number of inactive β centers in both cultivars, but the magnitude of change was higher in Guitang 49 than Guitang 28. Furthermore, in both cultivars, quantum efficiency of PSII, status of water splitting complex, and performance index were adversely affected by chilling, along with reduction in net photosynthesis rate and nighttime respiration and alterations in leaf optical properties. The extents of negative effect on these parameters were larger in Guitang 49 than in Guitang 28. These results reveal a clear differentiation in PSII heterogeneity between differentially chilling tolerant cultivars. Based on our studies, it is concluded that PSII heterogeneity can be used as an additional non-invasive and novel technique for evaluating any type of environmental stress in plants.

## Introduction

Sugarcane (*Saccharum officinarum* L.), a major C_4_ crop, is mainly cultivated in tropical and subtropical regions. It is an economically important crop having versatile uses, such as fodder, production of sugar, biofuel, paper, alcohol, etc. The highest sugar yield in sugarcane required mean optimum growth temperature between 25 and 30°C, whereas mean suboptimum growth temperature is <12°C–15°C, which causes reduction in growth rate, sugar yield, and biomass production (Ebrahim et al., 1998; Li and Yang, 2015; Li et al., 2015). Countries located in marginal tropical and subtropical regions face severe damage to sugarcane cultivation due to seasonal chilling temperature incidences (Selvarajan et al., 2018). For instance, China is in the third position among the top 10 sugarcane-producing countries with an annual sugarcane production of 104 mt (FAO, 2017). China encountered a chilling temperature incident during the year 2008, which caused 68% crop (sugarcane) loss in Guangxi province alone (Li and Yang, 2015; Li et al., 2015). On a global scale, occurrences of such deleterious chilling temperature incidents are predicted to be more frequent in the near future, due to climate change (IPCC, 2013).

Like other abiotic stress factors, chilling temperature mainly affects photosynthesis of plants and consequently causes reduced growth and yield (Sunoj et al., 2017; Liu et al., 2019). Previous studies have also reported that chilling temperature in marginal, tropical, and subtropical regions negatively affected photosynthesis of tropical plant species (Elsheery et al., 2008; Huang et al., 2010). Chilling temperature primarily has negative impact on electron transport between photosystems (PSII and PSI), carbohydrate metabolism-related enzymes [Rubisco, Rubisco activase, fructose 1,6-bisphosphatase (FBPase), and sedoheptulose 1,7-bisphosphatase (SBPase)], photorespiration, and stomatal response, which are different components related to photosynthesis. The impact of chilling temperature on any of the above components can result in photoinhibition of PSII and eventually affect functioning of PSI (Allen et al., 2000; Allen and Ort, 2001; Kadota et al., 2019).

The stability of photosystems under stress conditions is important to maintain the efficiency of light reaction and thereby proper functioning of dark reaction or *vice versa*. Among the photosystems, PSII is more sensitive to chilling temperature than PSI (Huang et al., 2010). Chlorophyll (Chl) *a* fluorescence can provide information about the functional status of PSII and is widely used for the study of impact of abiotic stresses in plants (Strasser et al., 2004; Sunoj et al., 2017; Mathur et al., 2019). Chl *a* fluorescence is not only used for investigating Chl transient curves but also used for studying PSII heterogeneity (Mathur et al., 2011). On the basis of functional and structural pattern, PSII heterogeneity is of two types, i.e., antenna size heterogeneity and reducing side heterogeneity (Anderson and Melis, 1983; Mathur and Jajoo, 2020). PSII antenna heterogeneity involves three types of reaction centers: α, β, and γ (Hsu et al., 1989). The dominant form, PSII α, is localized in the grana partition regions (Anderson and Melis, 1983) and is responsible for the majority of the water oxidation activity and plastoquinone reduction. These centers possess a Chl *a* core complex, an accessory Chl *a*–*b* light-harvesting inner antenna (LHC II-inner), and a peripheral antenna (LHC II-peripheral) containing a combined total of approximately 210–250 Chl *a* and Chl *b* molecules. PSII α is subpopulation of dimeric PSII. However, PSII β and γ subpopulations are found to be present in non-appressed region of thylakoid membranes and are considered as slow PSII centers (Anderson and Melis, 1983).

Another form of PSII heterogeneity is known as reducing side heterogeneity, which comprises reducing and non-reducing centers (Lavergne, 1982; Mathur et al., 2011). Some PSII centers are photochemically efficient but are unable to transfer electrons efficiently from electron acceptor Q_A_^–^ to secondary electron acceptor Q_B_. These centers are termed as PSII Q_B_-non-reducing centers. In these centers, Q_A_^–^ can be reoxidized by a back reaction with the donor side of PSII (Guenther et al., 1988). Q_B_-non-reducing differs from Q_B_-reducing center as these are incapable of reducing the PQ pool. PSII heterogeneity is one of the important strategies in plants to overcome various types of abiotic stresses such as high temperature and osmotic stresses (Mathur et al., 2011; Tomar et al., 2012). Under stress conditions, variation in antenna size and reducing side heterogeneity of PSII has been observed. PSII does this by altering the numbers of active and inactive reaction centers. The inactive reaction centers increase under stress conditions, and they revert back to active reaction centers when the stress is over. The changes in PSII heterogeneity have been found to be mostly reversible; however, under stress conditions such as extreme high temperature or salinity, the changes in PSII heterogeneity become irreversible, indicating the extreme severity of the stress (Mathur et al., 2011).

Guangxi province in China has major contribution to China’s sugar production, and chilling temperature incidences have led to great loss on economic status of sugarcane farmers as well as related industries in this province. Better understanding of the role of PSII heterogeneity can open windows for improving screening strategies for identifying the chilling tolerant sugarcane genotypes. Studies have been conducted to evaluate the impact of chilling stress on sugarcane, but an analysis of alterations in PSII heterogeneity on recently released popular cultivars of sugarcane under chilling stress has not been performed. Until now, most of the studies on PSII heterogeneity have been conducted on C_3_ crops and rarely on C_4_ crops (Mathur and Jajoo, 2020). This study aims to evaluate the role of PSII heterogeneity on two recently released cultivar of sugarcane a C_4_ crop, under chilling stress. C_3_ and C_4_ plants have different strategies to cope up with stress conditions. With this study, we want to establish the significance of measurement of PSII heterogeneity in C_4_ plants too.

## Materials and Methods

### Plant Materials and Growth Condition

The experiments were carried out in March 2019 using controlled environmental facility established at Guangxi University, Nanning, China (22.83°N, 108.28°E). Newly released, moderately chilling tolerant cultivar Guitang 49 and chilling tolerant cultivar Guitang 28 of sugarcane were selected to study the effect of chilling temperature on PSII heterogeneity, Chl *a* fluorescence, gas exchange (daytime photosynthesis and nighttime respiration), Chl index, and leaf optical properties. Both cultivars were developed by Guangxi Sugarcane Research Institute, Guangxi Academy of Agricultural Sciences, China. Plants were grown in pots with a dimension of width 33 cm × height 30 cm in greenhouse. After planting, the seedlings were allowed to grow for 75 days in greenhouse under ambient condition before shifting to growth chambers. Soil was used as a growing medium. Soil contents were as follows: organic matter = 19.65 g/kg, total *N* = 0.10%, total *P* = 0.04%, total K = 0.62%, hydrolytic *N* = 86 mg/g, available *P* = 4.7 mg/kg, available K = 74 mg/kg, soil pH = 5.7. Throughout the experiment period, seedlings were watered until the pot capacity to maintain the equal level of soil moisture. To maintain adequate nutrient status of soil, nitrogen (*N*; 70 mg/kg soil), potassium (K; 50 mg/kg soil), and phosphorus (P; 100 mg/kg soil) were applied to the soil, and Hogland’s solution (150 mL/pot) was applied once in 2 weeks (Hoagland and Arnon, 1950). During this period, seedlings were irrigated, fertilized, and managed to avoid pest and insects by following commercial cultivation practices. After germination, 1 g of systemic insecticide Marathon (1% imidacloprid, 1-[(6-chloro-3-pyridinyl) methyl]-*N*-nitro-2-imidazolidin-mine; OHP Inc., Mainland, PA, United States) was applied to each pot to avoid incidence of sucking pest.

After 75 days, seedlings at late tillering phase were transferred to a walk-in growth chamber (Conviron Model CMP 6050; Winnipeg, MB, Canada). The optimum day/night temperatures inside growth chamber were maintained at 25°C/15°C (Li and Yang, 2015; Li et al., 2015), relative humidity (RH) of 60%, and 12 h of photoperiod (6:00 am–6:00 pm) with photosynthetic active radiation (PAR) of 500 μmol m^–2^ s^–1^ at the plant canopy level using cool fluorescent lamps. Temperature, humidity, and light were maintained by inbuilt automated monitoring and controlling system of growth chamber. Except day/night temperatures, RH and PAR were maintained at the same level until the end of experimental period. A transition time of 5 h from day to night and *vice versa* was followed to replicate the diurnal temperature fluctuation under natural conditions. The seedlings were allowed to acclimate for 3 days under the optimum temperature condition. On 3 day of exposure to optimum temperature, all the physiological parameters were measured for Guitang 49 and Guitang 28. After this, the day/night temperature inside growth chamber was decreased to 10°C/5°C, and seedlings were allowed to expose to chilling temperature for 3 days, and measurements were done to record the changes in response to chilling treatment. After studying chilling stress, for recovery of seedlings, growth chamber temperature was set back to the optimum day/night temperatures and allowed seedlings to recover from the impact of chilling stress for 3 days.

### Chl *a* Fluorescence

Chl *a* fluorescence was measured using the Plant Efficiency Analyser (Handy PEA; Hansatech, Norfolk, England). Measurements were recorded from the middle part of the well-developed and completely opened leaves after 30 min of dark adaptation. The light was provided by light emitting diode array of 650 nm focused onto the leaf to provide homogeneous irradiance over the exposed area (*d* = 4 mm). A pulse (for 1 s) of illumination with 3,000 μmol (photon) m^–1^ s^–1^ was applied to the leaf to generate maximal fluorescence (F_m_ for all the treatments). Ten to 25 measurements were recorded from each treatment. The efficiency of water splitting and oxygen evolving complex is determined by Fv/Fo and Vk/Vj, where Vk = (F_300 μ s_ - Fo)/(Fm - Fo); Vj = (F_2 ms_ - Fo)/Fm - Fo) (Brestic et al., 2012). Fo represents the minimum fluorescence, when all PSII RCs are open, fluorescence intensity at 50 μs. RC/ABS represents density of active reaction centers per Chl molecule and/or Q_A_ reducing RCs per PSII antenna Chl (Brestic et al., 2012). The multiplicative parameters used to obtain performance index [PI_(total)_] was directly measured from PEA. The PI_(total)_ is an overall parameter calculated for conservation the energy of absorbed by PSII photons until the reduction of PSI end acceptors. The parameters are the density of reaction centers (RC/ABS); the parameter [Φ_Po_/(1-Φ_Po_)], where Φ_Po_ represents the maximum quantum yield of primary photochemistry; the parameter [ψ_Eo_/(1-ψ_Eo_)], where ψ_Eo_ represents the efficiency with which an electron moves into the electron transport chain further than Q_A_^–^; the parameter (δ_Ro_/(1-δ_Ro_), where δ_Ro_ represents the efficiency with which an electron from the intersystem electron carriers is transferred to reduce end electron acceptors at the PSI (Photosystem I) acceptor side. 1-Vj represents the probability (at time 0) that a trapped exciton moves an electron into the electron transport chain beyond Q_A_^–^ (Strasser et al., 2004; Brestic et al., 2012).

### PSII Heterogeneity

#### Reducing Side Heterogeneity

Q_B_-reducing and Q_B_-non-reducing centers were calculated using double hit (pulse) method as mentioned in Strasser and Tsimilli-Michael (1998) and Mathur et al. (2011). In this method, two fluorescence transients were induced by two subsequent pulses (each of 1-s duration). The first pulse was conducted after a dark period long enough to ensure the reopening of all reaction centers, followed by a second pulse. The duration of the dark interval between two hits was 500 ms. Q_B_-non-reducing centers were calculated by the following equation:

$$\mathrm{Vo}(\mathrm{Bo})\;=\;\left[(\mathrm{Fv}/\mathrm{Fm})-(Fv^{\prime }/Fm^{\prime })]/(\mathrm{Fv}/\mathrm{Fm}\right)$$

where Bo = relative amount of Q_B_-non-reducing PSII centers.

#### Antenna Heterogeneity

Antenna size heterogeneity was measured using DCMU [3-(3,4-dichlorophenyl)-1,1-dimethylurea], poisoning method. The detached leaves were put in DCMU solution overnight in complete darkness [DCMU concentration was 200 μM (Toth et al., 2005), and the solution contained 1% ethanol, which was used to dissolve the DCMU]. The leaves were removed from the DCMU solution (in darkness), wiped, and left in the air for ∼1 h to avoid possible effects of anaerobiosis. Antenna size heterogeneity was calculated in terms of percentage of PSII α, β, and γ centers as per Hsu et al. (1989) and Mathur et al. (2011). α, β, and γ centers were calculated from the complementary area growth curve (Melis and Homann, 1976). It involved the calculation of growth of normalized complementary area, defined by the fluorescence induction curve and the line parallel with the maximum level of fluorescence (Fm), with time. Kinetics of complementary area of the dark-adapted sample was fitted with three exponentials phases (corresponding to α, β, and γ).

### Leaf Optical Properties and Chl Index

Leaf optical properties (light reflectance, transmittance, and absorptance) were measured between 11:00 am and 12:00 pm using a miniature leaf spectrometer (CI-710; Camas, WA, United States). Chl index was measured using Chl meter (SPAD-502 Plus; Konica Minolta Inc., Japan).

### Leaf Gas Exchange

Leaf gas exchange parameters such as net photosynthesis rate (*P*_N_), stomatal conductance (*g*_s_), transpiration (*E*), intercellular CO_2_ (*C*i), and nighttime respiration were recorded using a portable photosynthesis system (Li-6400XT; LI-COR, Lincoln, NE, United States). The apparent carboxylation capacity (CE) was calculated from the ratio of *P*_N_ to *C*_i_ (Rymbai et al., 2014).

From each temperature condition and cultivar, a minimum of nine photosynthesis and night respiration measurements were recorded from the middle part of the selected leaves between 10:00 and 11:00 am [after >4 h of exposure to light (PAR of 500 μmol m^–2^ s^–1^)] and 10:00 and 11:00 pm (after >4 h of exposure to dark), respectively. While measuring photosynthesis, PAR was set to 500 μmol m^–2^ s^–1^ inside the leaf chamber of portable photosynthesis system, and zero PAR was set for night respiration. At the same time, CO_2_ concentration was set to 400 μmol mol^–1^ in the leaf chamber, which was common for measuring photosynthesis and night respiration. The block temperature of leaf chamber was adjusted to respective set temperature condition inside growth chamber. The flow rate for photosynthesis measurement was 500 μmol s^–1^ and was adjusted to 100 μmol s^–1^ for measuring night respiration to minimize fluctuations (Li-6400XT; Portable Photosynthesis System; Version 6; LI-COR, Lincoln, NE, United States; Sunoj et al., 2016). While measuring night respiration, adequate attention was taken to avoid exposure seedlings to PAR from any external sources and previous to measuring night respiration, it was further confirmed by measuring the light with quantum sensor on leaf chamber of portable photosynthesis system.

### Statistical and Data Analysis

Six biological replications per cultivar were used to record different traits under different temperature conditions. Analysis of variance (ANOVA) was performed using generalized linear model in SPSS (version 16; SPSS Inc., United States) to test the significance of differences in all measured parameters of cultivars under different temperature conditions. The mean values of each cultivar under different temperature conditions were compared using Duncan multiple-range test (DMRT), whereas mean values of both cultivars under individual temperature condition were compared using Student *t*-test. Data and graphs and for Chl *a* fluorescence and PSII heterogeneity were statistically analyzed using GraphPad Prism 5.01 (GraphPad Software, Inc., La Jolla, CA, United States) and Origin Pro8. To deduce information from the O-J-I-P transients, normalizations and computations were performed using the Biolyzer 4HP software, and Origin Pro8 was used for graphical presentation.

## Results and Discussion

Significant difference was observed in both cultivars for all the studied parameters such as PSII heterogeneity, Chl *a* fluorescence transient parameters, and gas exchange traits for optimum temperature, chilling stress and for recovery as well. Results showed that moderately chilling tolerant Guitang 49 showed slow recovery as compared to tolerant Guitang 28.

### Effect of Chilling Stress on Chl *a* Fluorescence

**FIGURE 1:**
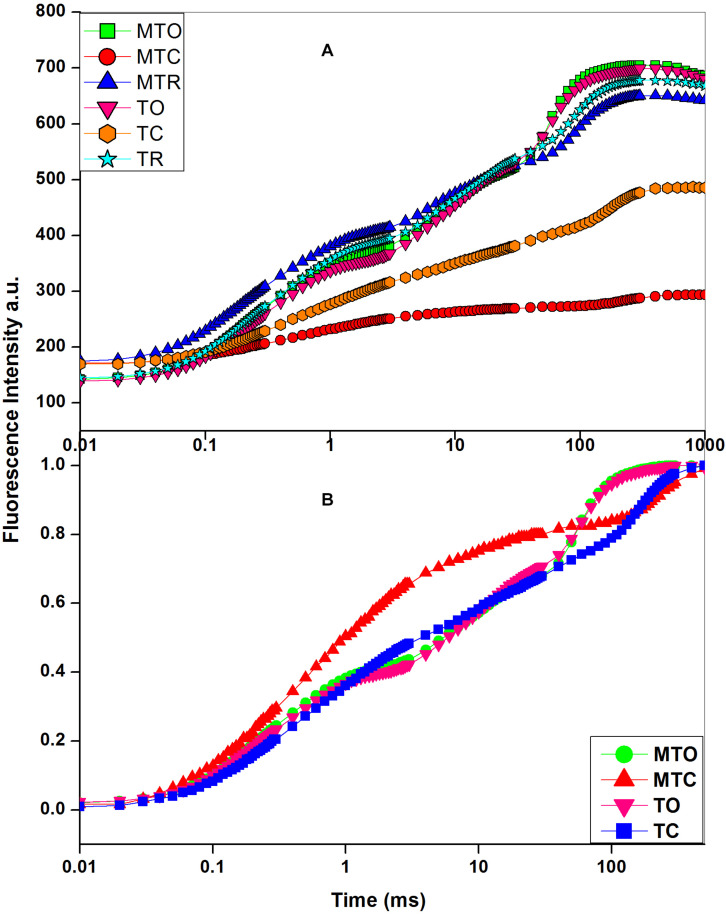
**(A)** Chlorophyll *a* fluorescence transient curves. **(B)** Double normalized chlorophyll *a* transient curve in sugarcane cultivars, under optimal, chilling, and recovery temperature conditions, 25°C/15°C (day/night optimum temperature), 10°C/5°C (after 3 days’ exposure of day/night chilling temperature), and 25°C/15°C (recovery at 3 days after experiencing day/night chilling temperature, respectively. MT, moderately tolerant cultivar (Guitang 49); T, tolerant cultivar (Guitang 28). The letters following MT or T such as O, C, and R denote the cultivars under the optimal and chilling temperatures and at recovery, respectively. a.u. represents arbitrary unit. Figure represents the average traces (*n* = 20) for MTO, MTC, MTR, TO, TC, and TR.

In OJIP transients (Figure 1), a distinct decline in J-P phase was obtained after chilling in both cultivars, but decline was prominent in Guitang 49. In Guitang 49, a decline in J-P phase was observed, indicating inhibition of electron transport through plastoquinone pool, while P phase was present in Guitang 28 (Figure 1A). The decline in J-P phase in Guitange 49 is clearly evident in the double-normalized figure (Figure 1B). Under chilling stress, quantum efficiency of PSII (Fv/Fm) decreased drastically in Guitang 49, indicating a decline in primary photochemistry (Table 1). Chilling stress decreased Fm and increased Fo (minimal fluorescence) (Table 1), which ultimately decreased Fv/Fm ratio. An increase in Fo may be probably because of the disconnection of PSII light-harvesting antennae from the PSII core complex (Brestic et al., 2012) due to chilling stress. A decline in maximal fluorescence was observed, which represented damage at the donor side of PSII (Table 1). Further, to confirm whether chilling stress had affected OEC and donor side of PSII, Vk/Vj was calculated for both cultivars (Table 1). The ratio of Vk/Vj decreased (Table 1) under chilling stress. The decline in Vk/Vj values suggested either a decrease in functional antenna size (Yusuf et al., 2010) or OEC inactivation (Kalachanis and Manetas, 2010; Brestic et al., 2012) due to chilling stress in both cultivars. The values for Vk/Vj were largely recovered in both cultivars. Furthermore, as a consequence of chilling stress, the electrons accumulated around plastoquinone (PQ) pool increasing the pool size but were unable to move electrons beyond Q_A_ and Q_B_ due to chilling stress (also indicted in later part of the manuscript), therefore causing a decrease in maximal fluorescence. This was also supported by a change in 1-Vj values. The ratios for 1-Vj decreased (Table 1), suggesting that chilling stress effects were observed not only on the donor side but also on the acceptor side of PSII (Brestic et al., 2012).

**TABLE 1:** Changes in chlorophyll *a* fluorescence parameters in sugarcane cultivars under different temperature conditions [25°C/15°C (OT; day/night optimum temperature); 10°C/5°C (CT; after 3 days’ exposure of day/night chilling temperature); and 25°C/15°C (RT; recovery at 3 days after experiencing day/night chilling temperature)].

	^a^Fo		^b^Fm		^c^Fv/Fm		^d^RC/ABS		^e^Fv/Fo		^f^PI_(total)_		^g^1-Vj		^h^Vk/Vj	
Cultivars (C)																

Temperature conditions (T)	MT	T	MT	T	MT	T	MT	T	MT	T	MT	T	MT	T	MT	T
25°C/15°C (OT)	130^c^ ± 13	126^bc^* ± 20	705^a^ ± 21	699^a^* ± 14	0.820^a^ ± 0.01	0.820^aNS^ ± 0.002	0.830^a^ ± 0.002	0.88^a^* ± 0.03	4.42^a^ ± 0.31	4.55^aNS^ ± 0.80	2.15^a^ ± 0.35	2.39^a^* ± 0.10	0.61^a^ ± 0.01	0.63^aNS^ ± 0.01	0.64^a^ ± 0.02	0.65^aNS^ ± 0.01
10°C/5°C (CT)	169^a^ ± 20	167^aNS^ ± 18	294^c^ ± 12	469b** ± 21	0.43^c^ ± 0.02	0.64** ± 0.03	0.350^c^ ± 0.002	0.781^ab^** ± 0.004	0.74^c^ ± 0.01	1.81^b^** ± 0.24	0.100^b^ ± 0.001	0.780^c^** ± 0.001	0.31^c^ ± 0.02	0.42^c^** ± 0.03	0.48^c^ ± 0.04	0.50^c^* ± 0.01
25°C/15°C (RT)	166^ab^ ± 11	134^b^** ± 11	651^b^ ± 21	678^a^* ± 17	0.750^b^ ± 0.01	0.800^a^* ± 0.01	0.630^b^ ± 0.002	0.791^ab^** ± 0.002	2.92^b^ ± 0.7	4.06^a^** ± 0.83	0.940^b^ ± 0.003	1.72b** ± 0.23	0.54^b^ ± 0.01	0.58^bNS^ ± 0.03	0.59^b^ ± 0.01	0.61^bNS^ ± 0.01
**Probability (*P*) values**																
C		<0.05		<0.01		<0.01		<0.01		<0.05		<0.01		<0.01		<0.01
T		<0.01		<0.01		<0.01		<0.01		<0.01		<0.01		<0.01		<0.01
C × T		<0.05		<0.01		<0.05		<0.01		<0.05		<0.05		<0.01		<0.01

MT, moderately tolerant cultivar (Guitang 49); T, tolerant cultivar (Guitang 28). Values following ± are standard error of respective mean values (n = 20). In ANOVA section, C, T, and C × T represent temperature conditions, cultivars, and interaction of C and T, respectively. ^a^Fo, minimal fluorescence; ^b^Fm, Maximum fluorescence; ^c^Fv/Fm, quantum efficiency of PSII; ^d^RC/ABS, reaction center per absorption; ^e^Fv/Fo, status of water splitting complex; ^f^PI_(total)_, performance index; ^g^1-Vj, the probability (at time 0) that a trapped exciton moves an electron into the electron transport chain beyond QA^–^; ^ h^Vk/Vj, status of OEC and functional antenna size. Mean values of cultivars followed by different letters are significantly different according to Duncan multiple-range test (DMRT; P < 0.01; the test was conducted independently for each cultivar). Mean values of both cultivars under individual temperature condition were compared using Student t-test; significance at *P < 0.05 and **P < 0.01, respectively. NS indicates non-statistically significant.

PI_total_ presents an overall view and health status of a plant (Strasser et al., 2004). (PI_total_) drastically decreased under chilling stress in both cultivars, with larger reduction in Guitang 49 than Guitang 28 (Table 1). This damping off in PI represented inefficiency of reaction centers and electron transport and also the negative down-regulation of primary photochemistry of PSII by chilling stress. After chilling treatment, the extent of recovery was greater in Guitang 49 as compared to Guitang 28 as the level of damage was also larger in the former.

Reaction center per absorbance (RC/ABS), i.e., the number of active Q_A_ reducing reaction centers per antenna unit (Brestic et al., 2012), was less affected in Guitang 28 (∼10%) as compared with Guitang 49 (∼57%) (Table 1). As the reaction centers of the former were active even under chilling condition, this could be one of the reasons for robustness of Guitang 28. Fv/Fo represents water splitting complex at the donor side of PSII and primary photochemistry. Because of chilling stress, Fv/Fo was decreased to nearly 83 and 60% in Guitang 49 and Guitang 28, respectively (Table 1), indicating damage at water splitting complex, resulting in reduced primary photochemistry. The plants were kept for recovery for 3 days. After 3 days of recovery, Guitang 28 recovered to 90% of the original value of the optimum temperature, whereas Guitang 49 recovered only 66% of the optimum temperature (Figure 1 and Table 1), indicating an enhancement in primary photochemistry and overall photosynthetic efficiency.

### Analysis of Antenna Size Heterogeneity, Leaf Optical Properties, and Chl Index Under Chilling Stress

**FIGURE 2:**
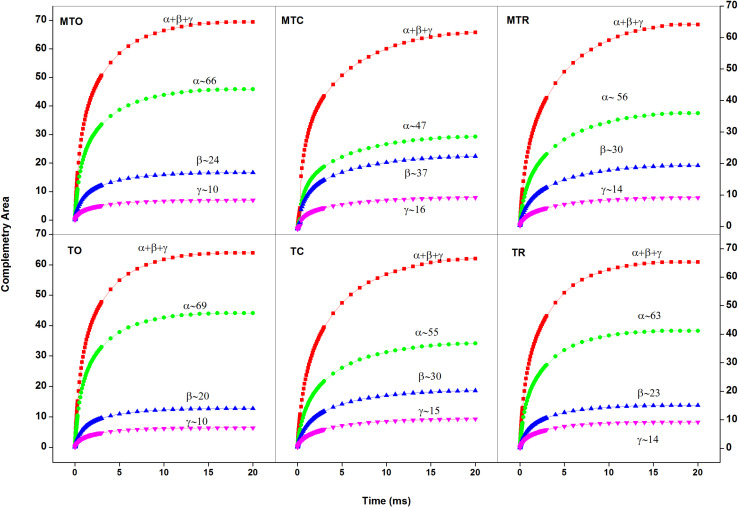
Complementary area growth curve showing percentage of PSIIα, β, and γ centers in sugarcane cultivars under different temperature conditions [25°C/15°C (day/night optimum temperature); 10°C/5°C (after 3 days’ exposure of day/night chilling temperature); and 25°C/15°C (recovery at 3 days after experiencing day/night chilling temperature)], where MT, moderately tolerant cultivar (Guitang 49); T, tolerant cultivar (Guitang 28). The letters following MT or T such as O, C, and R denote the cultivars under the optimal and chilling temperatures and at recovery, respectively. Figure represents the average traces (*n* = 10) for MTO, MTC, MTR, TO, TC, and TR.

Chilling stress caused a decrease in the number of active α centers and to a less extent increased active β centers (Figure 2) in both cultivars. PSII_α_ comprises Chl *a–*associated core complex, an accessory Chl *a–b* light-harvesting inner antenna (LHC II-inner), and a peripheral antenna (LHC II-peripheral) (Anderson and Melis, 1983). The decrease in the number of PSII_α_ may be a short-term response resulting in reorganization of the membrane with the disconnection of part of the antenna from PSII (Tikkanen and Aro, 2012). Another possibility is that under chilling stress, there is reorganization of PSII supercomplexes through monomerizations and migration, which often takes place under stress conditions (Bielczynski et al., 2016). Decrease in the number of PSIIα is accompanied with an increase in the number of PSII_β_ followed by the conversion into the smallest PSII_γ_ due to chilling stress. This also led to migration of β components from appressed to non-appressed region of the thylakoid membrane, resulting in an increase in the number of inactive centers. Further, after 3 days of recovery, compared with Guitang 49, Guitang 28 showed more recovery for antenna size heterogeneity.

Meanwhile, under chilling stress, leaf absorptance was decreased, and transmittance was increased in both cultivars (Table 2). However, reflectance of both cultivars exhibited contrasting trend. Guitang 28 showed lower transmittance and reflectance and higher absorptance under optimum and recovery conditions as compared to Guitang 49. But reflectance was increased in Guitang 28 when exposed to chilling stress, whereas transmittance was higher in Guitang 49. After 3 days of recovery, except transmittance, other optical properties were recovered from chilling stress (Table 2).

**TABLE 2:** Change in leaf optical properties (light reflectance, transmittance, and absorptance) and chlorophyll index in sugarcane cultivars under different temperature conditions [25°C/15°C (OT; day/night optimum temperature); 10°C/5°C (CT; after 3 days’ exposure of day/night chilling temperature); and 25°C/15°C (RT; recovery at 3 days after experiencing day/night chilling temperature)].

	Reflectance		Transmittance		Absorptance		Chlorophyll index (SPAD values)	
Temperature conditions (T)	Cultivars (C)							
	MT	T	MT	T	MT	T	MT	T
25°C/15°C (OT)	0.15^a^ ± 0.03	0.13^bNS^ ± 0.04	0.14^b^ ± 0.02	0.11^b^* ± 0.01	0.71^b^ ± 0.42	0.76^b^* ± 0.54	46.5^a^ ± 2.1	53.7^a^** ± 5.5
10°C/5°C (CT)	0.12^b^ ± 0.01	0.18^a^** ± 0.02	0.20^a^ ± 0.05	0.16^a^** ± 0.02	0.68^c^ ± 0.35	0.68^cNS^ ± 0.22	44.0^ab^ ± 6.3	51.0^ab^** ± 6.6
25°C/15°C (RT)	0.14^a^ ± 0.03	0.13^bNS^ ± 0.01	0.08^c^ ± 0.01	0.07^cNS^ ± 0.01	0.79^a^ ± 0.50	0.80^aNS^ ± 0.31	43.3^ab^ ± 5.1	51.6^ab^** ± 3.2
**Probability (*P*) values**								
C		<0.05		<0.05		<0.05		<0.01
T		<0.01		<0.01		<0.05		<0.01
C × T		<0.05		<0.05		<0.05		<0.05

MT, moderately tolerant cultivar (Guitang 49); T, tolerant cultivar (Guitang 28). Values following ± are standard error of respective mean values (n = 12 for optical properties and n = 18 for chlorophyll index). In ANOVA section, C, T, and C × T represent temperature conditions, cultivars, and interaction of C and T, respectively. Mean values of cultivars followed by different letters are significantly different according to Duncan multiple-range test (DMRT; P < 0.01; the test was conducted independently for each cultivar). Mean values of both cultivars under individual temperature condition were compared using Student t test; significance at *P < 0.05 and **P < 0.01, respectively. NS indicates non-statistically significant.

Under different stress conditions, changes in leaf optical properties at visible wavelengths (400–720 nm) arise because of variations in Chl content (Carter and Knapp, 2001; Brugger et al., 2019). Chl is the key absorber of light in leaf, and metabolic maladjustments under chilling stress modify the leaf Chl content (Bauerle et al., 2004; Sakaigaichi et al., 2019). A slight decrease was observed in Chl index of both cultivars under chilling stress and recovery conditions, while there was no significant variation in the magnitude of change from optimum temperature condition to chilling temperature and recovery conditions (Table 2). Previously, Tang et al. (2015) reported the higher degree of decrease in Chl index of sugarcane under a higher level of chilling stress (<4°C) for longer duration, which was attributed to the accelerated rate of degradation of Chl pigments, and the same trend was reported in sugarcane clones under <10°C as well (Sakaigaichi et al., 2019). The slight reduction in our study can be the initial stage of Chl degradation, and slower recovery rates specify the same. Even a slight variation in Chl content has higher impact on leaf absorptance, and that in turn affects the reflectance and transmittance (Carter and Knapp, 2001; Bauerle et al., 2004). This alteration of optical properties in the current study is more likely a result of a loss or degradation of Chl content.

### Analysis of Reducing Side Heterogeneity Under Chilling Stress

Reducing side heterogeneity decreased more in Guitang 49 as compared with Guitang 28 under chilling stress (Table 3). Reducing centers decreased by ∼37% in Guitang 49, whereas ∼20% in Guitang 28. The number of reducing centers decreased because chilling stress converted these reducing or active centers into inactive centers, and these centers were now unable to transport electron. Because of chilling stress, it might be possible that the active reducing centers migrated to the non-appressed part of thylakoid membrane from the appressed part (Anderson and Melis, 1983). In inactive centers, the electron transport from Q_A_ to PQ is nearly thousand times slower as compared with Q_B_ reducing centers. Under chilling conditions with increase in Q_B_ non-reducing center, a higher proportion of energy is diverted toward fluorescence and heat dissipation rather than being utilized in primary charge separation. As a result, increase in the proportion of Q_B_ non-reducing PSII centers increased the fluorescence emission at the O phase (Figure 1). This increase led to a linear decrease in Fv/Fm (Table 1; Zhu et al., 2005). After 3 days of recovery, Guitang 28 showed a higher rate of recovery as compared to Guitang 49.

**TABLE 3:** Percentage change in Q_B_ reducing and Q_B_ non-reducing centers in sugarcane cultivars under different temperature conditions [25°C/15°C (day/night optimum temperature); 10°C/5°C (after 3 days’ exposure of day/night chilling temperature); and 25°C/15°C (recovery at 3 days after experiencing day/night chilling temperature)].

	Percentage (%) of Q_B_ reducing centers		Percentage (%) of Q_B_ non-reducing centers	
Temperature conditions (T)	Cultivars (C)			
	MT	T	MT	T
25°C/15°C (OT)	81^a^ ± 4	84^a^* ± 12	19^c^ ± 2	16^c^* ± 1
10°C/5°C (CT)	51^c^ ± 9	67^c^** ± 4	49^a^ ± 9	33^a^** ± 5
25°C/15°C (RT)	68^b^ ± 11	71^b^* ± 7	32^b^ ± 1	29^b^* ± 2
**Probability (*P*) values**				
C		<0.05		<0.05
T		<0.01		<0.01
C × T		<0.05		<0.05

MT, moderately tolerant cultivar (Guitang 49); T, tolerant cultivar (Guitang 28). Values followed ± are standard error of respective mean values (n = 20). The letters following MT or T such as O, C, and R denote the cultivars under the optimal and chilling temperatures and at recovery, respectively. In ANOVA section, C, T, and C × T represent temperature conditions, cultivars, and interaction of C and T, respectively. Mean values of cultivars followed by different letters are significantly different according to Duncan multiple-range test (DMRT; P < 0.01; the test was conducted independently for each cultivar). Mean values of both cultivars under individual temperature condition were compared using Student t-test; significance at *P < 0.05 and **P < 0.01, respectively. NS indicates non-statistically significant.

### Effect of Chilling Stress on Gas Exchange

Under chilling stress, photosynthesis rate (*P*_N_), stomatal conductance (*g*_s_), transpiration (*E*), night respiration, and carboxylation capacity (*CE*) were significantly decreased in Guitang 49 as compared to Guitang 28. However, intercellular CO_2_ (*C*i) increased more in Guitang 49 (Table 4). Also, Guitang 49 showed delayed recovery of above traits. Chilling stress causes partial closure of stomata because of simultaneous engagement of stomatal and non-stomatal regulations (Allen et al., 2000; Allen and Ort, 2001; Huang et al., 2016; Arena and Vitale, 2018), which resulted in decreased P_N_, g_s_, E, and CE and increase in Ci of sugarcane cultivars in the current study.

**TABLE 4:** Change in photosynthetic gas exchange traits in sugarcane cultivars under different temperature conditions [25°C/15°C (OT; day/night optimum temperature); 10°C/5°C (CT; after 3 days’ exposure of day/night chilling temperature); and 25°C/15°C (RT; recovery at 3 days after experiencing day/night chilling temperature)].

	Nighttime respiration (μmol m^–2^ s^–1^)		Net photosynthesis (*P*_N_; μmol m^–2^ s^–1^)		Stomatal conductance (*g*_s_; mol m^–2^ s^–1^)		Intercellular CO_2_ (*C*i; ppm)		Transpiration (*E*; mmol m^–2^ s^–1^)		Carboxylation capacity (*CE*)	
Cultivars												
Temperature conditions (T)	MT	T	MT	T	MT	T	MT	T	MT	T	MT	T
25°C/15°C (OT)	1.57^a^ ± 0.14	2.49^a^** ± 0.27	17.09^a^ ± 2.31	16.63^a^* ± 0.76	0.10^a^ ± 0.03	0.12^a^* ± 0.01	111.5^c^ ± 28.7	148.11^a^** ± 32.0	1.13^a^ ± 0.30	1.01^c^* ± 0.08	0.15^a^ ± 0.04	0.12^a^* ± 0.03
10°C/5°C (CT)	1.39^b^ ± 0.27	2.21^a^** ± 0.35	4.41^c^ ± 0.74	12.21^c^** ± 1.26	0.03^c^ ± 0.01	0.08^b^** ± 0.02	163.5^a^ ± 21.7	126.47^b^** ± 27.9	0.51^c^ ± 0.11	1.20^b^** ± 0.16	0.03^c^ ± 0.01	0.10^b^** ± 0.03
25°C/15°C (RT)	0.75^c^ ± 0.12	1.07^b^** ± 0.14	9.81^b^ ± 1.00	14.60^b^** ± 1.33	0.07^b^ ± 0.01	0.09^b^* ± 0.01	140.7^b^ ± 31.1	114.96**^c^ ± 20.4	0.95^b^ ± 0.16	1.30^a^** ± 0.14	0.07^b^ ± 0.01	0.13^a^* ± 0.02
**Probability (*P*) values**												
C		<0.01		<0.01		<0.05		<0.01		<0.01		<0.01
T		<0.01		<0.01		<0.01		<0.01		<0.01		<0.01
C × T		<0.05		<0.05		<0.05		<0.05		<0.05		<0.05

MT, moderately tolerant cultivar (Guitang 49); T, tolerant cultivar (Guitang 28). Values followed by ± are standard error of respective mean values (n = 9). In ANOVA section, C, T, and C × T represent temperature conditions, cultivars, and interaction of C and T, respectively. Mean values of cultivars followed by different letters are significantly different according to Duncan multiple-range test (DMRT; P < 0.01; the test was conducted independently for each cultivar). Mean values of both cultivars under individual temperature condition were compared using Student t-test; significance at *P < 0.05 and **P < 0.01, respectively. NS indicates non-statistically significant.

Non-stomatal limitation occurs by the deactivation of enzymes associated with carbon fixation and related cascade of physiological and biochemical maladjustments, which increases *C*i under chilling stress (Zhang and Scheller, 2004). This trend was observed in Guitang 49, and it was further confirmed by the higher decrease of *P*_N_ and CE (Table 4). *CE* is the ratio between *P*_N_ and *C*i; lower *CE* values indicate higher *C*i due to the lower carbon fixation (Rymbai et al., 2014). Meanwhile, an opposite trend was observed in Guitang 28. From this trend, it can be speculated that the enzymes related to carbon fixation was least affected in Guitang 28, which leads to the minor decrease in *P*_N_ and *CE* (Table 4). Correspondingly, reduced *g*_s_ is an indicator of stomatal limitation induced by chilling stress (Allen and Ort, 2001). Reduction in g_s_ and E indicated that stomata of Guitang 49 were highly sensitive and affected with chilling stress. Similarly, response of Chl *a* fluorescence transients (Fv_/_Fm, Fv/Fo, and PI) also points toward lower tolerance of Guitang 49 and higher tolerance of Guitang 28 (Table 1).

Simultaneously, low rate of nighttime respiration was also a factor for lower chilling tolerance of Guitang 49 (Table 4). A higher level of chilling injuries to thylakoid membrane and photosystems of Guitang 49, as evident from the changes antenna size and reducing side heterogeneity and Chl *a* fluorescence transients values, caused reduction in photosynthesis, which subsequently delayed recovery of Guitang 49 (Table 1). Particularly, such circumstances occur because of reduction of maintenance respiration (Sunoj et al., 2016, 2020), which can cause slower maintenance of chilling injuries. On the other hand, ability of Guitang 28 to sustain higher nighttime respiration was an advantage over Guitang 49 under chilling stress by repairing the chilling injuries, maintaining a certain level of Fv/Fm, Fv/Fo, PI, and photosynthesis and was thereby supported for faster recovery of Guitang 28 (Tables 1, 4).

Photosynthesis and respiration (day and night time) are correspondingly significant for the carbon trade-off and growth (Sunoj et al., 2020). In plants, rather than energy cost for ion uptake and transport and growth, nighttime respiration has a significant role in maintenance of injuries, which occurs because of oxidative stress triggered by overproduction and accumulation of reactive oxygen species under optimum and diverse biotic and abiotic stress conditions (Djanaguiraman et al., 2013; Sperling et al., 2015). The balance between functions of nighttime respiration, specifically growth and maintenance, differs with the type and intensity of stress factors. Depending on the magnitude of damage caused by stress, more resources, approximately 30–70% of photosynthetically generated photosynthates (carbohydrates) instead of seldom used fatty acids, are channelized and utilized for the maintenance rather than growth (Djanaguiraman et al., 2013; Sunoj et al., 2016, 2020).

Such a kind of allocation of photosynthates via higher night respiration was reported to cause reduction in total biomass accumulation in maize and sorghum, but under long-term exposure to high-temperature stress condition (40 days) (Sunoj et al., 2016, 2020). However, in the current study, with short-term chilling stress exposure (3 days), relatively higher respiration than Guitang 49 contributed to the chilling tolerance of Guitang 28 by the maintenance of chilling injuries, and such trend can be considered as an advantage to confront sudden chilling incidence, which last only for a few days in natural condition. By virtue of adaptation in certain tolerant plant species to different abiotic stress factors, the magnitude of nighttime respiration varies as compared to the other susceptible plant species. Under chilling stress, nighttime respiration was reported to reduce in some plant species such as lettuce, tomato, and soybean (Frantz et al., 2004), which is similar to response of sugarcane cultivars used in the current study.

## Conclusion

The present study revealed that chilling stress adversely affected PSII heterogeneity along with Chl *a* fluorescence transients, gas exchange (daytime photosynthesis and night respiration), leaf optical properties, and Chl index. Results indicated that the impact of chilling stress was high in moderately chilling tolerant Guitang 49 as compared to tolerant Guitang 28. A dramatic decline was obtained in maximal fluorescence, indicating damage at the donor side of PSII, and reflected in decreased quantum efficiency of PSII under chilling stress. Reaction centers of tolerant cultivar Guitang 28 were not much affected. Both cultivars showed distinguishable difference for reducing side and antenna size heterogeneity under chilling stress. Under chilling stress, Q_B_ non-reducing centers increased, due to conversion of active reducing centers into inactive ones. Chilling stress led to an increased number of PSII_β_ and PSII_γ_ centers followed by a decrease in PSII_α_ center. Under chilling stress, reducing side heterogeneity and antenna size heterogeneity were downregulated more in Guitang 49 as compared to Guitang 28. Higher photosynthesis and nighttime respiration have also been proven to be a supporting factor for Guitang 28 to survive under chilling stress. The magnitude of recovery varied according to cultivar and extent of damage. This is the first ever study that has focused on PSII heterogeneity under chilling stress on the C_4_ crop sugarcane and proven an important role of PSII heterogeneity in sugarcane genotype under chilling stress, with connection to other physiological traits. Based on the present study, we conclude that PSII heterogeneity can be used as an additional non-invasive and novel technique for evaluating environmental stress in plants.

## Data Availability Statement

The original contributions presented in the study are included in the article/supplementary material, further inquiries can be directed to the corresponding author/s.

## Author Contributions

SM, VS, and NE designed the study. SM and VS performed the experiments and analyzed the data and wrote the manuscript. AJ, K-FC, and VR edited the manuscript. All authors contributed to the article and approved the submitted version.

## Conflict of Interest

The authors declare that the research was conducted in the absence of any commercial or financial relationships that could be construed as a potential conflict of interest.

## Abbreviations

Chl: chlorophyll
Ci: intercellular CO_2_
E: transpiration
ET: electron transport
Fm: maximal fluorescence
Fv/Fm: quantum efficiency of PSII
g_s_: stomatal conductance
PI: performance index
P_N_: Net photosynthesis
RC/ABS: Reaction center per absorption.
